# Residual Neural Processing of Musical Sound Features in Adult Cochlear Implant Users

**DOI:** 10.3389/fnhum.2014.00181

**Published:** 2014-04-03

**Authors:** Lydia Timm, Peter Vuust, Elvira Brattico, Deepashri Agrawal, Stefan Debener, Andreas Büchner, Reinhard Dengler, Matthias Wittfoth

**Affiliations:** ^1^Department of Neurology, Hannover Medical School, Hannover, Germany; ^2^Center of Functionally Integrative Neuroscience, Aarhus University, Aarhus, Denmark; ^3^Royal Academy of Music, Aarhus, Denmark; ^4^Brain and Mind Laboratory, Department of Biomedical Engineering and Biomedical Science, Aalto University School of Science, Aalto, Finland; ^5^Cognitive Brain Research Unit, Institute of Behavioral Sciences, University of Helsinki, Helsinki, Finland; ^6^Neuropsychology Laboratory, Department of Psychology, Carl von Ossietzky University of Oldenburg, Oldenburg, Germany; ^7^Cluster of Excellence Hearing4All, Oldenburg/Hannover, Germany; ^8^Department of Otolaryngology, Hannover Medical School, Hannover, Germany.; ^9^Department of Clinical Psychology and Sexual Medicine, Hannover Medical School, Hannover, Germany

**Keywords:** cochlear implant, auditory evoked potentials, mismatch negativity, music multi-feature paradigm, music perception

## Abstract

Auditory processing in general and music perception in particular are hampered in adult cochlear implant (CI) users. To examine the residual music perception skills and their underlying neural correlates in CI users implanted in adolescence or adulthood, we conducted an electrophysiological and behavioral study comparing adult CI users with normal-hearing age-matched controls (NH controls). We used a newly developed musical multi-feature paradigm, which makes it possible to test automatic auditory discrimination of six different types of sound feature changes inserted within a musical enriched setting lasting only 20 min. The presentation of stimuli did not require the participants’ attention, allowing the study of the early automatic stage of feature processing in the auditory cortex. For the CI users, we obtained mismatch negativity (MMN) brain responses to five feature changes but not to changes of rhythm, whereas we obtained MMNs for all the feature changes in the NH controls. Furthermore, the MMNs to deviants of pitch of CI users were reduced in amplitude and later than those of NH controls for changes of pitch and guitar timber. No other group differences in MMN parameters were found to changes in intensity and saxophone timber. Furthermore, the MMNs in CI users reflected the behavioral scores from a respective discrimination task and were correlated with patients’ age and speech intelligibility. Our results suggest that even though CI users are not performing at the same level as NH controls in neural discrimination of pitch-based features, they do possess potential neural abilities for music processing. However, CI users showed a disrupted ability to automatically discriminate rhythmic changes compared with controls. The current behavioral and MMN findings highlight the residual neural skills for music processing even in CI users who have been implanted in adolescence or adulthood. **Highlights**:

- Automatic brain responses to musical feature changes reflect the limitations of central auditory processing in adult Cochlear Implant users.
- The brains of adult CI users automatically process sound features changes even when inserted in a musical context.
- CI users show disrupted automatic discriminatory abilities for rhythm in the brain.
- Our fast paradigm demonstrate residual musical abilities in the brains of adult CI users giving hope for their future rehabilitation.

## Introduction

A cochlear implant (CI) is a device, which can restore hearing in patients with severe and profound sensori-neural hearing loss. The outer and middle ear is bypassed with a microphone and a speech processor, which converts the acoustical signals into electric pulses. These pulses are brought into the cochlear nerve via the transmitter coil and thus stimulate directly the hearing nerve fibers. Despite the limitations of their implant, most CI users are able to derive information for speech intelligibility, depending on the age when the device has been implanted. Usually younger implantees (implantation age <4 years) reach better levels of speech understanding than older implantees as long as the critical time window for speech acquisition is considered (Kral and O’Donoghue, 2010). However, for post-lingually deafened CI users the levels of speech understanding are depending on factors such as: duration of implant use, amount of training, and rehabilitation as well as psychological factors like: personal acceptance of the implant and environmental reactions (Gfeller et al., 2008; Driscoll et al., 2009). Since the CI was mainly created as a prosthesis to enhance speech perception, music perception remains comparably poor (Koelsch et al., 2004; Gfeller et al., 2006; Cooper et al., 2008; Limb and Rubinstein, 2012). These differences arise mainly because of the missing spectral fine structure information, which is not well processed by the current CIs (McDermott, 2004). Behavioral measures of CI users’ auditory capabilities compared to NH controls, however, imply a number of confounding factors such as fluctuations in attention, differences in familiarity with and motivation in relation to performing auditory tasks, and so on. In the electrophysiology lab, the mismatch negativity (MMN) brain response is instead elicited while the subject is performing a task unrelated to the sounds, allowing the study of automatic auditory skills in the brain (Alho et al., 1998; Brattico et al., 2006; Näätänen et al., 2012). Even though the number of published experiments so far is very small, the MMN has emerged as a reliable marker for CI users’ ability to accurately discriminate stimuli without the trade-off of subjective behavioral responses (Kraus et al., 1993; Lonka et al., 2004; Kelly et al., 2005; Sandmann et al., 2010; Zhang et al., 2011; Torppa et al., 2012).

The MMN is a component of the auditory event-related potential (ERP) recorded with electroencephalography (EEG) in response to sound features (such as pitch, timber, and intensity), or abstract rules (such as musical scale relations) deviating from those of a predictable auditory environment (Näätänen, 1992; Näätänen et al., 2001, 2011a). The MMN is sensitive to discrimination learning (Näätänen et al., 1993) and hereby to auditory and musical competence (Vuust et al., 2005; Vuust and Roepstorff, 2008; Brattico et al., 2009; Tervaniemi, 2009), being it elicited even by small changes in stimulus features at a level near just-noticeable difference thresholds (Näätänen et al., 2007) and provides an objective measure of central auditory processing functions. Traditionally, the MMN is obtained by using the oddball paradigm, which includes a repetitive sound and an infrequent change in one feature of the sound, such as its frequency or duration or timber. With a stimulus trial lasting, for instance, about one second, the oddball paradigm would require about 15 min of sound repetitions to reach an acceptable signal-to-noise ratio necessary to obtain averaged brain responses to a single sound feature change. Hence, to obtain MMN responses to several feature changes, several hours of recordings would be needed. Obviously, that is not affordable with a clinical population (and difficult even with healthy subjects); consequently, most MMN and, more broadly, ERP papers using traditional oddball paradigms provide brain responses to a single feature change (e.g., Näätänen et al., 2012). That, however, is unsatisfactory because the neuroauditory profile of the subjects is not accurate when only one feature is studied. For instance, the evolving of schizophrenia seems to be reflected in the MMN to frequency chances whereas the genetic aspects of the disease may be more closely associated with the deficient MMN to duration changes [for a review, see Näätänen and Kahkonen (2009)]. Indeed, the first version of the multi-feature paradigm, introduced by Näätänen et al. (2004) was later applied by Sandmann et al. (2010) to demonstrate that MMNs to changes in a repeated sound occurring 50% of the times may be elicited even in CI users. In addition, Torppa et al. (2012) have demonstrated how a new multi-feature change detection paradigm can be used in order to demonstrate cortical processing of musical sound in young CI users. They have found significant (although in some cases reduced) neural responses to several feature changes in children using CIs, which did differ from those of the NH control group only in response to changes in musical instrument, sound duration, and gap but not for other sound features, demonstrating the potentials of music intervention in CI children. The possibility to measure MMNs to several sound feature changes in a laboratory session lasting less than 20 min opens thus new opportunities for basic research with young children and for opening new interventional avenues.

Recently, Vuust et al. (2011) have introduced a new fast musical multi-feature paradigm that tests sound feature deviations in a complex auditory setting resembling music. This paradigm can be used as a tool of objective assessment of music-expertise neural skills in normal-hearing listeners (Vuust et al., 2011, 2012a,b). In the musical multi-feature paradigm, deviant sound features (such as pitch, timber, intensity, and rhythm) are embedded in the “Alberti bass,” where three different pitches alternate in a four-note pattern changing over the 12 keys. The stimuli therefore provide a more musical context than the original multi-feature paradigm in which one sound feature alternated with a deviant one (cf. Torppa et al., 2012). Indeed, the musical multi-feature paradigm has evidenced differences between different kinds of musicians, which were closely related to the style-specific aspects of the music practiced (Vuust et al., 2012a).

Based on the correlation between musical expertise and the amplitude of the MMN obtained in a normal-hearing population including musicians (Vuust et al., 2012a), we hypothesized that adult CI users would show distinct MMNs for musical features with different magnitudes of deviations depending on the feature and the characteristics of their corrected hearing. Compared to NH controls, we anticipated longer latencies in the CI users as well as smaller MMN amplitudes, indexing their impaired music processing. However, without previous studies measuring brain processing of several features in a musical context in adult CI users, we could hypothesize a difference between musical feature processing in CI users, without any more specific expectation on which direction this difference would be evidenced.

## Materials and Methods

### Participants

Twelve adult right-handed CI users (age range in years: 21–56, mean: 43.5, SD: 9.97) and 12 age- and sex-matched, right-handed participants with normal-hearing ability (age range in years: 21–57, mean: 43.3, SD: 11.09) were included. Prior to the experiment, all CI users had been using their implant for at least 12 months. All CI users were implanted during adulthood except one participant who received the implant at age 13. Moreover, all CI users were post-lingually deafened with the duration of profound deafness not exceeding 18 years (years of profound deafness: 5.93, SD: 6.24) (please see Table 1 for detailed patient demographics). Additionally, their hearing abilities exceeded 20% as assessed by the Freiburger monosyllabic words test in quiet environment, a standard German speech intelligibility test in which participants repeat monosyllabic words presented at a level of 65 dB. All experimental procedures were approved by the local ethics committee and the study protocol conformed to the Declaration of Helsinki. Participants gave written informed consent before data collection and received monetary compensation for their time.

**Table 1:** **Patient demographics**.

Subject	Age	Sex	Implant type	Duration of profound	Age at implantation	Etiology	Freiburger monosyllabic	Implanted
				deafness (years)	(years)		in quiet (%)	ear
P1	34	F	AB Clarion CII	1.59	26	Sudden	90	Right
P2	55	M	AB HiRes 90 K	6.76	53	Progressive	65	Right
P3	56	F	AB HiRes 90 K	10.3	52	Genetic	90	Right
P4	44	F	Nucleus RE 24	17	39	Measles	85	Right
P5	43	M	AB HiRes 90 K	0.34	42	Progressive	90	Left
P6	40	M	Medel SONATA	<0.2	35	Hypoxia	65	Right
P7	50	M	Nucleus RE 24	<0.2	47	Otosclerosis	45	Right
P8	46	F	Nucleus RE 24	3.17	40	Genetic	90	Right
P9	35	F	Nucleus RE 24	5.67	29	Progressive	90	Right
P10	21	F	Nucleus RE 24	7	13	Genetic	65	Left
P11	48	F	Nucleus RE 24	17.75	43	Mumps	90	right
P12	51	M	Medel SONATA	1.25	46	Sudden	80	Left

### Stimuli

The auditory stimuli in the present experiment were similar to the musical multi-feature paradigm developed by Vuust et al. (2011), with only small adaptations due to the characteristics of the CI patient group. Unlike the oddball paradigm, the multi-feature paradigm allows us to record auditory evoked potential (AEP) responses to many auditory feature deviations in a relatively short time and with a comparably good signal-to-noise ratio. Instead of a usual stimulus probability (80% standards; 20% deviants), our current musical multi-feature paradigm allows each “standard” to be followed by a “deviant” resulting in an equal probability of standards and deviants.

The musical multi-feature paradigm is an extension of the “optimal paradigm” (Näätänen et al., 2004) but with a richer musical context and higher complexity obtained by presenting standards and deviants within an “Alberti bass” configuration. This configuration is commonly used in the Western musical culture in both classical and improvisational music genres. For the present study, we presented this musical 4-tone pattern, with a key change between F-major, G-major, A-major, or C-major on every sixth measure. The original paradigm by Vuust et al. (2011) was adapted to the CI patient group by limiting the amount of key changes, in order to meet the average frequency range of the CI user devices. The keys were kept in the middle register of a piano with the bass note between *F*3 and *E*4, while their order was pseudo-randomized; each key was repeated six times during the experiment. In addition, whenever a key change occurred, the standard pattern was repeated six times in order to facilitate the difference between standard and deviant pattern in the presence of a key change. Those standard patterns occurring after key change were omitted from the average.

**Figure 1:**
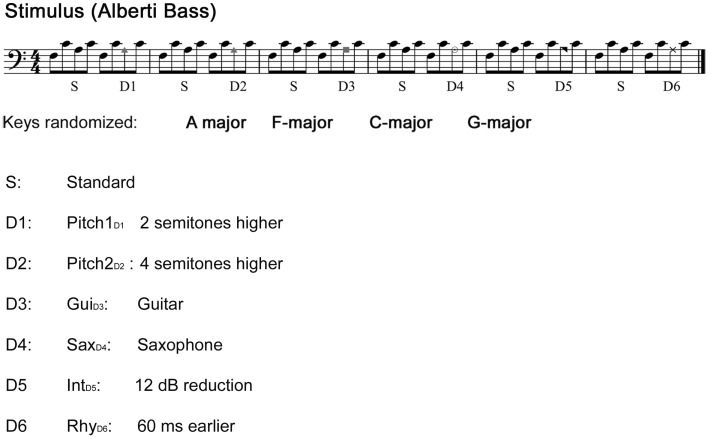
**Stimuli**. The main stimulus: “Alberti bass” patterns alternating between standard sequence and a deviant sequence played with a piano sound. Patterns were periodically transposed to four different keys with an interval of six bars. Each tone was 200 ms in duration, with an inter-stimulus-interval of 5 ms, yielding a tempo of approximately 140 beats/min. Comparisons were made between the third note of the standard sequence and the third note of the deviant sequence.

Sound stimuli were generated using the sample sounds of an acoustic piano (Wizoo) from the software sampler “Halion” in Cubase (Steinberg Media Technologies GmbH). Deviant patterns were similar to the standards, except that the third tone of the pattern was modified with Pro Tools (Pro Tools 7.4, Avid) as illustrated in Figure 1.

The first pitch deviant (Pitch1_D1_) was created by exchanging the third tone of the Alberti pattern with a sound, which was two semitones higher. The second pitch deviant (Pitch2_D2_) was created in the same manner using a substitute four semitones higher. Note that Pitch2_D2_ produces both a pitch and contour violation, whereas Pitch1_D1_ only produces a pitch violation. The two timber deviants were created by exchanging the third note for either a guitar (Gui_D3_) or a saxophone (Sax_D4_) sound (both timber deviants were normalized in loudness according to the standard pattern). The intensity deviant (Int_D5_) was generated by reducing the original loudness of the third tone by 12 dB, whereas the rhythm deviant (Rhy_D6_) was created by anticipating the third note by 60 ms. Each single note was presented in stereo (44,100 Hz sample frequency), and with a duration of 200 ms and with a 5 ms inter-stimulus-interval form the previous tone (except the rhythm deviants). The deviants occurred always in the same fixed order as depicted in Figure 1. The stimuli were presented with Presentation software (Neurobehavioral Systems). The total duration of the experiment was 20 min.

### Procedure

#### EEG experiment

Upon arriving to the lab, participants signed the consent form and were subsequently prepared for EEG recordings. The EEG was recorded from 30 scalp channels using active electrodes (Acticap, Brain Products, Munich, Germany) placed according to the 10–20 system (Klem et al., 1999) with a BrainAmp (Brain Products, Munich, Germany). For the CI users, three to six channels mainly from the temporal (T12/T8) to the occipital electrodes (P08) had to be unattached due to interferences with the implant transmission coil (channels range: 3–6, mean: 3, SD: 1). Two electrodes were attached to record the EOG (below and at the outer canthus of the right eye). The reference electrode was attached to the nose-tip and was used as the common reference. Sampling rate was 250 Hz, the data were analog filtered (0.1–80 Hz), and electrode impedances were kept below 10 kΩ. During the EEG recordings, participants were comfortably seated in a shielded chamber and passively listened to the auditory sequences via loudspeakers positioned on their left and right side with an angle of 45°. Loudness was kept at a sound pressure level of 60 dB. All participants watched a silenced documentary throughout the whole experimental procedure.

#### Behavioral experiment

After the EEG recordings, all participants performed a discrimination task to measure a behavioral index of their auditory discrimination accuracy. In this three alternative choice task, participants were presented with the same four-tone pattern as used in the previous EEG experiment. The pattern was presented three times in a row (3 × 4-pattern), twice in the standard condition and once with in a deviant condition. The deviating pattern could occur either on the first, the second, or the last position in the presentation of the 3 × 4-pattern. All deviant conditions were presented equally often and were repeated 10 times in random order. Participants were instructed to press a corresponding key (1, 2, 3) indicating at which position the deviating pattern had occurred. Hit rates of CI users and NH controls were analyzed and averaged across the six deviant conditions.

### Data analysis

Electroencephalography data were analyzed in the MATLAB (Mathworks, Nattick, MA, USA) environment using EEGLAB 9.0.5.6b (Delorme and Makeig, 2004). Data were filtered offline using a FIR filter with the lower edge of the frequency pass band at 1 Hz and a higher edge of the frequency pass band at 30 Hz. The recordings were screened for infrequent or un-stereotyped artifacts using an inbuilt probability function (pop_jointprob) with a threshold of three standard deviations (Debener et al., 2008). After performing an Infomax independent component analysis (ICA), ocular and cardiac artifacts were identified using the CORRMAP plug-in (Viola et al., 2009) and removed from the data. Artifacts caused by electrical interference of the CI were identified with respect to their independent components (ICs) (Debener et al., 2008; Viola et al., 2011, 2012). Evaluation of whether an IC was artifact driven was determined by (i) visual inspection of IC scalp projection (e.g., centroid of activity on the implanted side), (ii) whether on and offset of the AEP component were in phase with stimulus on and offset, or (iii) whether the activity power spectrum of the IC showed a periodic-like spectral distribution in the frequency domains up to 20 Hz (Torppa et al., 2012). Consequentially, ICs found to reflect an artifact induced by the implant were removed from the data.

For the CI users, the missing channels were spherically interpolated with respect to the neighboring channels to enable voltage topographic maps. Following ICA-based artifact attenuation, data were segmented in 100 ms pre-stimulus and 400 ms post-stimulus epochs. After baseline correction (−100 to 0 ms), single subject averages of the six types of deviant stimuli as well for the standard stimuli were conducted. Single-subject MMN latencies and amplitudes were measured by subtracting the AEP waveform of the deviant from the standard waveform resulting in six difference-waves. For the MMN quantification, group- and deviant-specific time windows of 40 ms were chosen from the respective grand-average MMN peak amplitude. MMN amplitude voltages for all electrodes were then calculated as the mean amplitude within these 40 ms time windows (see Table 2 for time windows). In line with previous studies (Näätänen et al., 2007; Duncan et al., 2009) reporting that the largest negative MMN peak is typically obtained at Fz, MMN significance analysis against the zero baseline was carried out on electrode Fz. Since the mastoids were not accessible in all CI users, we chose P08 to evaluate possible polarity reversals of the MMN response (Sandmann et al., 2010).

**Table 2:** **Amplitudes and latencies of the MMN in response to different musical features for both groups**.

Deviant	Interval (ms)	CI users				NH controls				
		Amplitude mean (μV)	*t*	SD	latency (ms) (SD)	Interval (ms)	Amplitude mean (μV)	*t*	SD	latency (ms) (SD)
Pitch1_D1_	180–220	−0.61	−2.81*	0.75	202 (15.3)	136–176	−1.80	−7.45**	0.83	148 (14.4)
Pitch2_D2_	180–220	−0.71	−3.11*	0.79	206 (19.7)	136–176	−2.48	−7.48**	1.15	148 (12.3)
Guit_D3_	140–180	−1.38	−5.07**	0.94	165 (14.1)	120–160	−2.72	−7.76**	1.23	134 (14.0)
Sax_D4_	140–180	−1.10	−5.22**	0.73	169 (14.7)	140–180	−1.71	−4.53**	1.31	165 (13.0)
Int_D5_	138–178	−1.49	−4.77**	1.08	150 (17.3)	138–178	−1.39	−4.22**	1.14	162 (16.4)
Rhy_D6_	128–168	−0.24	−1.41	0.74	143 (11.1)	128–168	−1.91	−6.50**	1.01	140 (14.1)

*p = 0.01; **p < 0.001.

### Statistics

Two-tailed *t*-tests were carried out for all six deviant categories in both groups to ascertain that MMN amplitudes differed significantly from zero. A repeated measure ANOVA with within-subject factor deviation (five levels: Pitch1_D1_, Pitch2_D2_, Gui_D3_, Sax_D4_, Int_D5_) and Group as between-group factor was computed for MMN latencies. For further statistical analysis, the effects of feature deviation on the MMN amplitudes and scalp distributions in terms of frontal and central electrodes as well as group-specific differences were calculated on a subset of electrodes (*F*3, Fz, *F*4, C3, Cz, C4). A repeated measures ANOVA was performed on the MMN mean amplitudes and latencies. Within-subject factors were Deviation (five levels: Pitch1_D1_, Pitch2_D2_, Gui_D3_, Sax_D4_, Int_D5_), Frontality (two levels: F-line, C-line), and Laterality (left, middle, or right), while Group was a between-subject factor. Effects of electrode factors alone are not reported as meaningless with respect of the hypothesis tested concerning group differences (they only reflect the scalp topography of the MMN). A Greenhouse-Geisser correction was applied when necessary, and will be indicated in the following section with epsilon values; degrees of freedom will be presented uncorrected. *Post hoc t*-tests were used to reveal group-specific differences.

## Results

### MMN amplitudes

**Figure 2:**
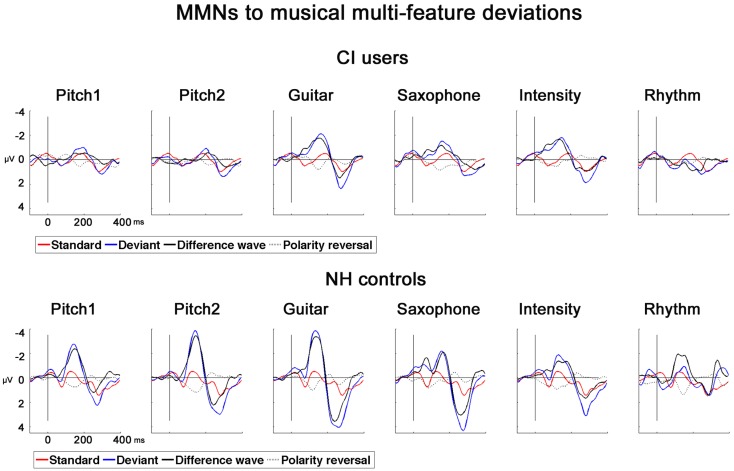
**MMNs to musical multi-feature deviations in CI users and NH controls**. Grand-average AEPs for CI users and NH controls for six types of deviations recorded at Fz. Standard (red), deviant (blue), difference wave (black), polarity reversal was obtained at P08 (dotted line).

In NH controls, the fast multi-feature paradigm elicited significant MMNs in all the six feature deviants whereas in CI users significant MMNs were found for all but the Rhy_D6_ (see Table 2). For the MMN amplitudes, we found a significant main effect of Group (*F*_1,22_ = 8.57; *p* = 0.008), deriving from overall diminished MMN in CI users compared to NH controls (mean value for combined MMNs as measured on Fz: CI users: −0.92 μV, SD: 0.88; NH controls: −2.00 μV, SD: 1.11). We also obtained significant within-subject effects of Deviation (*F*_4,88_ = 4.57; *p* < 0.001) (see Table 2). Furthermore, we found a significant interaction Deviation × Group (*F*_4,88_ = 3.86; *p* = 0.008). *Post hoc t*-tests for amplitude at *F*z with respect to deviation showed the largest differences between the two groups for the Pitch1_D1_ (*t* = 3.64; *p* = 0.001) and Pitch2_D2_ (*t* = 4.39; *p* < 0.001) deviations. A significant difference was also found for the Gui_D3_ with smaller amplitudes in the CI users than in NH controls (*t* = 3.03; *p* = 0.006). We found no significant differences for the MMN amplitudes to saxophone and intensity between CI users and NH controls (Sax_D4_: *t* = 1.4, *p* = 0.17; Int_D5_: *t* = 0.20, *p* = 0.83). MMN amplitude for Rhythm_D6_ differed significantly between CI users and NH controls (*t* = 4.57, *p* < 0.001) (please see Figure 2 for MMNs to musical multi-feature deviations).

**Figure 3:**
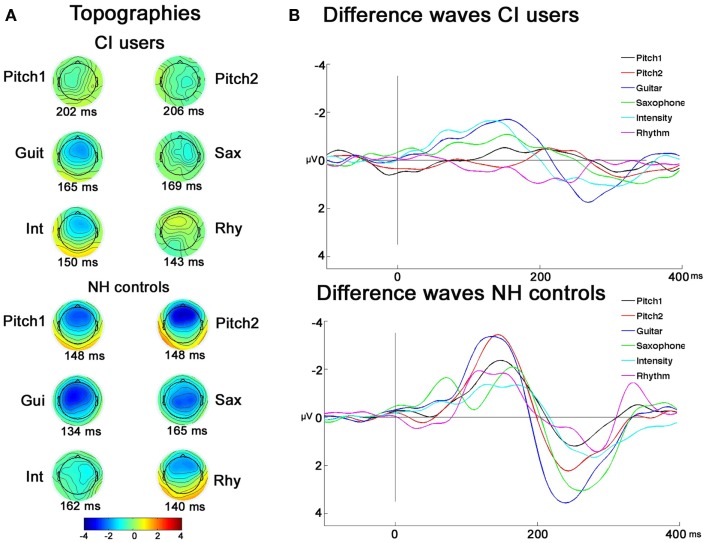
**Topographies and grand-average difference-waves of CI users and NH controls**. **(A)** EEG voltage isopotential maps of the difference between the responses to deviants and standards averaged in an interval of ±20 ms around maximal peak amplitudes. **(B)** Grand-average difference-waves of CI users and NH controls.

As illustrated in Figure 3, and indicated with *post hoc* paired *t*-tests the topography maps show that the MMNs of CI users were differently lateralized than those of the NH controls. This was testified also by the significant interactions between the between-subject factor and the two electrode factors: Laterality × Group (*F*_2,44_ = 5.20; *p* = 0.02), Frontality × Laterality × Group (*F*_2,44_ = 10.74; *p* = 0.001), and Frontality × Deviation × Group (*F*_4,88_ = 5.48; *p* = 0.004). Further investigating these interactions, planned *t*-tests showed that significant MMN lateralization was obtained for feature deviations Pitch1_D1_ (comparing *F*3 < *F*4: *t* = 3.32, *p* = 0.007) and Gui_D3_ (*F*3 < *F*4: *t* = 2.33, *p* = 0.040), whereas no significant differences for *F*-line vs. *C*-line were observed for the different feature deviations (all *p* > 0.6) in CI users. In the NH controls, both pitch deviants showed a more frontal (Pitch1_D1_ *F*4 > *C*4: *t* = 2.49, *p* = 0.030; Pitch2_D2_ *F*4 > *C*4: *t* = 4.94, *p* < 0.001) and rightwards lateralization (Pitch1_D1_ *F*4 > *F*3: *t* = 7.83, *p* < 0.001; Pitch2_D2_ *F*4 > *F*3: *t* = 3.51, *p* = 0.005). The MMN to feature deviation Gui_D3_ showed strongest amplitude on the C-line, with no significant lateralization effect (all *p* > 0.061).

### MMN latencies

The MMN latencies were modulated by the six feature deviations in both groups as tested with repeated measures ANOVA in a general linear Model and showed significant within-subject effect for Deviation (*F*_4,88_ = 13.75, *p* < 0.001) as well as an interaction Deviation × Group (*F*_4, 88_ = 22.16, *p* < 0.001). Furthermore a significant main effect of Group was found for the MMN latencies (*F*_1, 22_ = 125.42, *p* < 0.001). The two MMNs with the longest latency in the CI users were elicited by the two pitch deviants and differed significantly from the two pitch MMN latencies of the NH controls (Pitch1_D1_: *t* = 8.74; *p* < 0.001; Pitch2_D2_: *t* = 8.50; *p* < 0.001). The shortest MMN latency for the NH group was obtained for the Gui_D3_: this latency differed significantly from the one observed in CI users (*t* = 5.32; *p* < 0.001). Comparable to the results of the MMN amplitudes, we found no group-specific differences for the Sax_D4_ MMN latency (*t* = 0.645, *p* = 0.52) or the Int_D5_ MMN latency (*t* = 1.78, *p* = 0.88). The Rhy_D6_ MMN, was found for the NH controls only (see Table 2 for detailed latency and amplitudes measures).

### Behavioral experiment

All subjects showed a high accuracy with above-chance hit rates. We found lower hit rates for CI users compared to NHs in most feature deviation categories, including Pitch1_D1_ (*t* = −2.69, *p* = 0.013), Pitch2_D2_ (*t* = 2.46, *p* = 0.022), Gui_D3_ (*t* = 2.86, *p* = 0.009), and the deviation Int_D5_ (*t* = 2.45, *p* = 0.22), whereas the groups did not differ for the Sax_D4_ (*t* = 0.684, *p* = 0.50), or Rhy_D6_ (*t* = 0.01, *p* = 1.0) deviations (see Table 3 for Hit rates).

**Table 3:** **Hit rates of CI users and NH controls**.

	CI user hit rate		NH controls hit rate	
	(%)	SD	(%)	SD
Pitch1_D1_	65	2.42	87	0.90
Pitch2_D2_	74	3.07	98	0.40
Gui_D3_	85	1.12	97	0.64
Sax_D4_	92	1.20	96	1.20
Int_D5_	68	3.20	92	1.42
Rhy_D6_	77	2.40	76	2.01

### Correlations between MMN and behavioral or demographic measures

**Figure 4:**
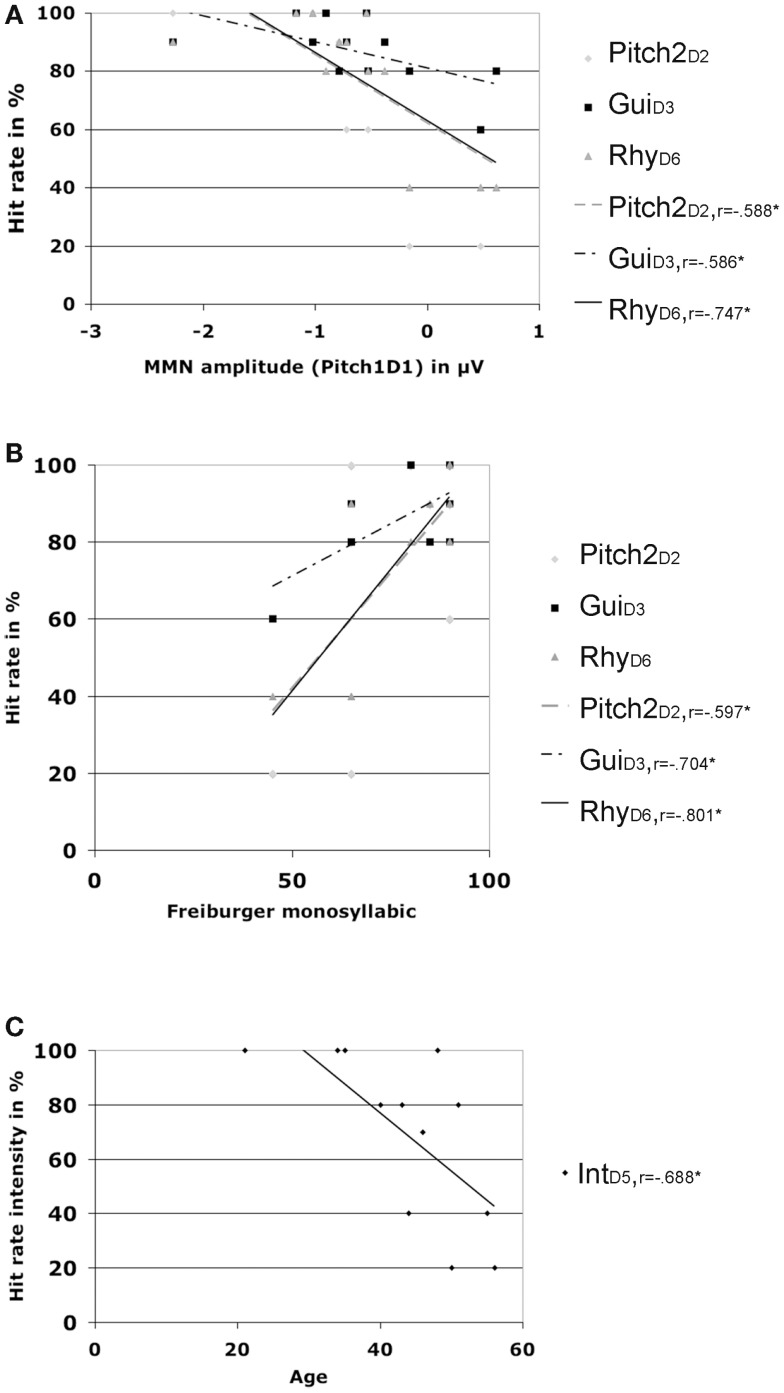
**Hit rates and correlations**. **(A)** Correlations of different hit rates with MMN amplitude for deviation Pitch1 in CI users. **(B)** Correlations of different hit rates with the Freiburger monosyllabic speech scores in CI users. **(C)** Correlations of hit rates for intensity with age in CI users.

Additional correlations for the CI users group only, including MMN amplitudes at Fz, patient demographics, and hit rates showed significant positive correlations for the Freiburger speech score and hit rates for Pitch2_D2_ (*r* = 0.597, *p* = 0.04), Gui_D3_(*r* = 0.704, *p* = 0.011), and Rhy_D6_(*r* = 0.801, *p* = 0.002) (please see Figure 4). The same hit rates were also significantly negatively correlated (e.g., the higher the hit rate the larger the MMN amplitude) with the MMN amplitude for feature deviation Pitch1_D1_ (Pitch2_D2_: *r* = −0.588, *p* = 0.044), Gui_D3_ (*r* = −0.586, *p* = 0.045), and Rhy_D6_ (*r* = −0.747, *p* = 0.005).

Age was negatively correlated with the hit rate for Int_D5_ (*r* = −0.688, *p* = 0.013) and the MMN latency for feature deviation Pitch1_D1_ (*r* = −0.619, *p* = 0.032) with older CI users showing prolonged latencies for the pitch MNN (see Figure 2).

## Discussion

Electroencephalography studies with CI users yield challenges regarding recording, analysis and comparison with NH controls. Due to the implant itself fewer electrodes may be used, which results in a higher amount of topographical interpolated channels. In addition, the implant interferences with the EEG signal require a careful inspection and understanding of the origins of the CI artifact in order to be able to visualize and interpret the resulting evoked potentials of interest. Nevertheless, our results show evidence for CI users’ processing of prominent sound features embedded in a complex sound context. CI users in our study had five robust MMNs out of six for sound features formerly described as difficult for these subjects to perceive. We observed significant differences between CI users and NH controls for the MMN amplitude and latencies depending on the feature deviation, especially for the two pitch deviations. The timber deviant to saxophone as well as the intensity deviant elicited similar MMNs in both groups. CI users did not elicit a significant MMN for the rhythm feature even in a complex musical context, which might be explained by the relatively small magnitude of the rhythm deviation within a complex auditory context. In sum, we here extend the findings of earlier MMN studies (Ponton and Don, 1995; Sandmann et al., 2010; Zhang et al., 2011; Torppa et al., 2012), showing that CI users may be able to process musical features such as pitch and intensity even in a complex music-like context. Furthermore, the differences in the MMN scalp distributions and latencies between the different deviant types observed in the present suggest that partially separate neural populations process and store distinct auditory sensory memory traces for different sound features, such as pitch, timber, and intensity (Caclin et al., 2006; Näätänen et al., 2011b). Hemispheric asymmetries between CI users and NH controls for AEPs have been shown earlier by studies indicating a topographical (e.g., more ipsilateral) displacement due to the implantation (Sandmann et al., 2009; Gordon et al., 2010).

### Pitch

The findings of the Pitch1_D1_ in CI users indicate the capability of CI users to perceive differences as small as two semitones. However, less neural efficiency for pitch processing was observed with CI users as evidenced by their diminished MMN amplitudes and lower hit rates to both pitch deviants compared to controls. Especially under consideration of the correlation with the Freiburger speech scores, the pitch results indicate a dependency between the perception of small pitch differences and good speech perception. This extends the results of Torppa et al. (2012), who found that small pitch deviations might be sufficiently salient thus eliciting a MMN. While in their study young CI users were implanted early in life in our study adult CI users were implanted significantly later in life. In Torppa et al.’s study, children early implanted with a CI showed adequately and equally good processing of pitch when compared to NH control children for deviations of three to four semitones of repeated piano tones without any musical context or minimal acoustic variation. In our study, we elicited MMN in adult CI users who were mainly implanted late in life in response to a pitch deviation as little as two semitones, inserted in a music-like context. The findings indicate that the automatic neural processing of pitch [as indexed by the MMN (Näätänen et al., 2011a) is not limited to the often-referred five to seven semitones, when tested behaviorally (Gfeller et al., 2002; Donnelly et al., 2009)]. We found a robust MMN in CI users for the second pitch deviation with four semitones. The threshold of 2–4 semitones elicits a MMN in CI users is considerably good. Recently Lonka et al. (2013) presented similar findings on how the MMN in adult CI users to quasi four semitones (3200 Hz deviants to 4000 Hz standards) is robust and enhanced over the measurement time of 2.5 years.

Behavioral studies, which indicated pitch thresholds of at least five to seven semitones in CI users, often involve judgments of the direction of pitch differences (Gfeller et al., 2002; Drennan and Rubinstein, 2008). Our findings, on the contrary, reflect that neural automatic detection of a pitch change within a complex pitch pattern, takes place even with smaller deviations. Similar findings were reported by Peretz et al. (2009), who obtained a significant MMN to small pitch changes inserted in a complex melody context in patients with congenital amusia, despite no conscious awareness of those changes. The musical richness of context in our study and the previous one on congenital amusics might provide additional cues enabling sound-processing impaired subjects to at least neurally process feature changes. Since the MMN is an index of pre-attentive processing, however, this neural detection may not be sufficient for participants to make clear behavioral discriminations. This explanation is also supported by Leal et al. (2003) who described the differences between pitch discrimination and pitch identification abilities in adult CI users and the impaired prerequisite for the latter to detect the direction of the pitch change. These findings are, nevertheless, potentially important because they hint at the possibility of rehabilitation even in adult CI users who were implanted later in life, thanks to the presence of residual auditory discrimination capabilities in the brain.

### Timber

Both timber feature deviations (e.g., guitar and saxophone) elicited MMNs in CI users and NH controls. This corroborates earlier findings by Koelsch et al. (2004) showing significant MMNs for timbers differing from the standard piano sound in adult CI users. However, these timber deviants were implemented in a less musical setting than the one used in the current study, thus allowing less generalization of the findings to everyday life situations involving perception of complex auditory scenes.

Behavioral timber discrimination accuracy has been shown to be reflected by the MMN response to timber changes (Näätänen et al., 2007). The timber of an instrument is mainly defined by its temporal and spectral envelope. The gross temporal envelope and the sound onset are comparably good perceived by CI users, whereas the spectral envelope and especially the fine structure are partly missing (Drennan and Rubinstein, 2008; Heng et al., 2011). This might explain the comparable morphologies between the CI users and NH controls in the difference-waves for the two timber deviations, as well as the reduced MMN specifically to the guitar deviant. The guitar as a plugged string instrument has a sharper attack time, and therefore a steeper envelope compared to the slower, by air-excited saxophone. Again, one needs to differentiate between the acoustic change mechanism underlying the two MMNs in our experiment and the general timber identification abilities in adult and experienced CI users. These behavioral identification abilities are hampered depending on the target instrument of the identification task, musical training and a high inter-individual variability (Galvin et al., 2009). This hampered neural and behavioral timber abilities in CI users may be also driven by the fact that the required spectro-temporal fine structure, necessary to differentiate between timbers, is not fully provided by the current CI decoding strategies (Timm et al., 2012). The general consequence of such limitations in the CI device is a perceptual difficulty with complex sound environments (Moore, 2003).

### Intensity

Although hit rates for intensity differed significantly between groups, we found no group differences in MMN amplitudes or latencies. Instead, the intensity deviation showed the most comparable MMN morphologies between groups, along with the timber deviations. This is not surprising since intensity is usually well implemented in CI users. It is, however, plausible that CI users would be more uncertain about what they hear in general, and therefore behaviorally perform worse than NH despite the apparent similarity between the neural responses between the groups on this sound feature. This assumption is further supported by our findings of the negative correlation between the intensity hit rate and the CI users’ age. However, the amplitude range of the MMN in our adult CI users group was remarkably large compared to earlier studies (Sandmann et al., 2010; Torppa et al., 2012) and fosters the reliability of the current musical multi-feature paradigm.

### Rhythm

In music, changes in sound duration are necessary in order to be able to detect changes in rhythm and tempo. Interestingly, the rhythm deviant did not elicit a significant MMN in the CI users. Behavioral studies have shown that the rhythm perception is working well for adult CI users (Limb, 2006; Drennan and Rubinstein, 2008). However, the complexity and lack of attention toward the auditory stimuli in our experiment may have driven the lack of MMN to rhythm feature deviations, as already indicated by the low behavioral hit rate for this feature. This may give rise to the question, whether the behavioral rhythm tests, currently used with CI users within their rehabilitation training, give reliable results about their musical rhythm perception. It may rather be that simple clapping or single note rhythms are more easily perceivable with a CI, whereas rhythm nuances embedded in a complex auditory scene are more difficult to extract. This argument is corroborated by the relative minimal rhythm deviation of 60 ms used in our study, since various studies have indicated that adult CI users with a longer duration of profound deafness have difficulties in more complex rhythm discriminations with small rhythmic changes (Leal et al., 2003; Kong et al., 2004). Future studies should focus on the ability of adult CI users to understand and appreciate musical expression based on rhythmic and temporal variations.

### Summary

Our findings extend the insight on the neural abilities for musical feature processing in adult CI users who were implanted after childhood. Particularly, we showed that by using a music-like stimulation paradigm, CI users’ brains are able to extract more information from sound than previously reported, as indexed by the distinct MMNs to several musical features. This indicates the existence of residual feature encoding abilities in adult CI users. The musical multi-feature paradigm with which we tested these perceptual abilities is advantageously short and musically enriched compared to previous music-related MMN studies. Within 20 min, we were able to test for six types of deviations embedded in an ecologic musical setting. Our findings imply that it might be necessary to work with realistic stimulus changes in order to capture residual auditory processing skills. In turn, the neural processing of deviations in rhythm was seemingly more difficult in the present paradigm, thus explaining the previously reported differences in our study between behavioral data and AEPs as shown here in relation to the rhythm deviant.

The multi-feature paradigm implemented here may be adopted for clinical routine as it may give objective data of the capability of current implants in an everyday-like listening condition. However, to meet this goal future research in AEP method needs to reach sensitivity at the single subject level to enhance reliability of individual multi-attribute profiles of sound discrimination abilities. Further experiments should include a more parametric approach toward the single deviant categories leading to a more pronounced MMN and specific information about a magnitude of the deviance effect (Horvath et al., 2008; Näätänen, 2009). Additionally, differences between uni- and bi-lateral CI users could be tested giving more information concerning the lateralization of the MMN. This paradigm might also be suitable for auditory brainstem responses. Therefore, experiments including patients with an auditory brainstem implant are warranted, since there is evidence that the novelty detection reflected by the MMN might be driven by much earlier processes of deviant detection encoding mechanisms (Slabu et al., 2012).

## Conflict of Interest Statement

The authors declare that the research was conducted in the absence of any commercial or financial relationships that could be construed as a potential conflict of interest. The Review Editor Kimmo Alho declares that, despite being affiliated to the same institution as author Elvira Brattico, the review process was handled objectively and no conflict of interest exists.
